# Role of Cereal Secondary Metabolites Involved in Mediating the Outcome of Plant-Pathogen Interactions

**DOI:** 10.3390/metabo1010064

**Published:** 2011-12-15

**Authors:** Lauren A. Du Fall, Peter S. Solomon

**Affiliations:** Research School of Biology, College of Medicine, Biology and Environment, The Australian National University, ACT, 0200, Australia

**Keywords:** secondary metabolite, plant defence, pathogen, cereal, metabolism, benzoxazinoid, isoprenoid, terpene, flavonoid, cyanogenic glycoside, saponin

## Abstract

Cereal crops such as wheat, rice and barley underpin the staple diet for human consumption globally. A multitude of threats to stable and secure yields of these crops exist including from losses caused by pathogens, particularly fungal. Plants have evolved complex mechanisms to resist pathogens including programmed cell death responses, the release of pathogenicity-related proteins and oxidative bursts. Another such mechanism is the synthesis and release of secondary metabolites toxic to potential pathogens. Several classes of these compounds have been identified and their anti-fungal properties demonstrated. However the lack of suitable analytical techniques has hampered the progress of identifying and exploiting more of these novel metabolites. In this review, we summarise the role of the secondary metabolites in cereal crop diseases and briefly touch on the analytical techniques that hold the key to unlocking their potential in reducing yield losses.

## 1. Introduction

Eight major cereal crops including wheat, rice, barley, oat, rye, corn, sorghum and millet make up two-thirds of the worlds food supply [1]. Estimates list approximately 2.5 billion tonnes of cereals were produced in 2009, steadily growing from 800 million tonnes in the 1960's [2]. Biotic stresses, such as those caused by fungal pathogens, represent the greatest threat to global cereal production. For example, an epidemic of rice blast disease caused disastrous crop losses across China in the 1980's affecting up to 12% of the its rice acreage [3]. Fusarium head blight (scab; FHB) has historically been responsible for extensive crop losses throughout the world ranging from 15%–50% of wheat, barley and oat crops [4]. Rust fungus is also a significant pathogen of cereals causing losses of 0.73–1.73 million tonnes in India and Pakistan during 1972 and 1973 [1]. These are but a handful of many such examples.

Taking into account the vast number of potential plant pathogens that exist, the actual amount of disease is relatively small. This is attributable to an intricate array of defence mechanisms plants have evolved over time as a necessity to survive their immobile nature. Typically, disease is avoided when a host plant recognises the presence of a pathogen. This recognition activates various plant defence responses including phytoalexin production, primary metabolite signalling, production of reactive oxygen species, protease and chitinase production, cross-linking of cell wall polymers, production of pathogenesis related (PR) proteins and the hypersensitive response, which leads to localised cell death [5]. Physical defence mechanisms are also crucial in pathogen attack namely solidifying of cell walls with lignin, polymerisation and crosslinking also to strengthen cell walls and the presence of cuticular waxes. For a review on plant defence responses see [6].

Plants synthesise a diverse range of secondary metabolites active in defence against a wide variety of pathogens [7]. These secondary metabolites offer a survival advantage to the plant during pathogen attack but are generally considered non-essential for basic plant metabolism (Dixon, 2001). These metabolites have various roles such as feeding deterrents, allelopathic compounds and antimicrobial agents [8] and are either constitutively produced (phytoanticipans) or pathogen/stress induced compounds (phytoalexins) [9,10].

In recent years, substantial advances have been made in discovering and characterising secondary metabolites from both plant and animal sources. Significant technological advancements in high throughput and mass spectrometry (MS) have evolved a new research discipline called metabolomics -the study of small molecules in biological systems. A number of techniques are used for high throughput analysis of an extensive range of structurally and chemically diverse metabolites. Gas Chromatography-Mass Spectrometry (GC-MS) is utilised for analysis of polar metabolites following chemical derivatisation and volatiles using headspace analysis. Liquid Chromatography-Mass Spectrometry (LC-MS) is capable of analysing a range of polar and semi-polar compounds for which no chemical derivatisation is required. Nuclear Magnetic Resonance (NMR) Spectroscopy and Fourier Transform Infrared (FTIR) Spectroscopy are also utilised to structurally characterise small molecules however due to a combination of cost and complexity of the resulting data they are not as common as GC and LC-MS. These techniques enable identification and quantification of metabolites, which through carefully designed biological experiments, can be utilised to unravel the complex metabolite responses of plants to pathogens. An advantage of these metabolomics approaches over genomic and proteomic approaches is the ability to determine the exact metabolic state of the plant after pathogen infection.

This review will describe secondary metabolites involved in mediating the outcome of plant-pathogen interactions in cereals. Secondary metabolites will be discussed in the context of chemical class rather than their roles as phytoanticipan or phytoalexins as a number of compounds fall into both these categories in different species. These secondary metabolites offer tremendous potential for plant breeding and metabolic engineering in agriculture to aid in controlling existing disease losses [11,12].

## 2. Benzoxazinoids

**Figure 1.**
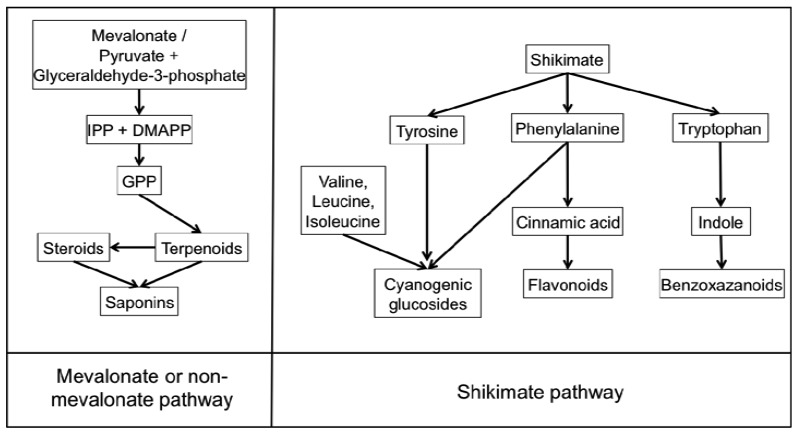
Simplified diagram illustrating the biosynthetic pathways of the discussed plant secondary metabolites involved in pathogen defence. Cyanogenic glucosides, flavonoids and benzoxazanoids are all synthesised from the aromatic amino acids derived from shikimate. Saponins and terpenoids are synthesised via the melavonate or non-melavonate pathway in plants, which occur in the cytoplasm and plastids of plants respectively.

Benzoxazinoids (Bxs) are a class of secondary metabolites widely distributed in cereals discovered in the 1950's and since found to have a range of biological roles including alleopathy, resistance to insects and defence against pathogens [13-15]. Bxs are synthesised from the amino acid tryptophan in the shikimate pathway (Figure 1). They are present in maize; wheat, rye and certain wild barley species however have not been found in cultivated barley varieties, oat or rice [15,16]. These compounds are found in all parts of the plants but are present at higher levels in younger leaves [15]. Bxs are stored in an inactive glucoside form in plant vacuoles or plastids to avoid toxicity to the plant itself; they undergo enzymatic and chemical degradation upon tissue disruption to form the active benzoxazinoid [16,17]. The mechanism by which these compounds exert phytotoxic activity may be due to: their mutagenic effects on DNA, ability to react with amino acids and perhaps therefore disrupt proteins [18].

The most common Bx in rye and wild barley is 2,4-dihydroxy-2*H*-1,4-benzoxazin-3(4*H*)-one (DIBOA) and in maize and wheat is 2,4-dihydroxy-7-methoxy-2*H*-1,4-benzoxazin-3(4*H*)-one (DIMBOA) (Table 1). Bxs are also involved in plant defence against pathogenic fungi that cause very little tissue disruption [19] suggesting other methods of Bx-mediated resistance. Ahmad *et al.* [20], investigated the role of Bxs in resistance of maize to the necrotrophic fungus *Setosphaeria turtica* at stages prior to tissue disruption. They found that Bxs accumulate at the highest concentration in apoplastic leaf extracts and are critical for basal resistance against *S. turtica*. Bxs therefore have roles as defence metabolites as well as a defence regulatory signal in maize. Recently, a number of new Bx derivatives were identified using Ultra Performance LC-MS/MS [21]. The authors identified double hexose derivatised metabolites of the three Bxs DIBOA, HBOA (2-(2-hydroxy-1,4(2*H*)-benzoxazin-3(4*H*)-on)-β-D-glucopyranoside and DIMBOA in whole grain rye and wheat; however not in oat or barley. The location of the hexose moieties on the Bx structure, the presence of these compounds in other parts of the plant and the role of these double hexose derivatised Bxs in plant resistance to pathogens is yet to be ascertained.

**Table 1.** Diagram illustrating the structures of a number of plant secondary metabolites belonging to the major classes of defence compounds discussed.

**Compound class**		
**Benzoxazanoids**	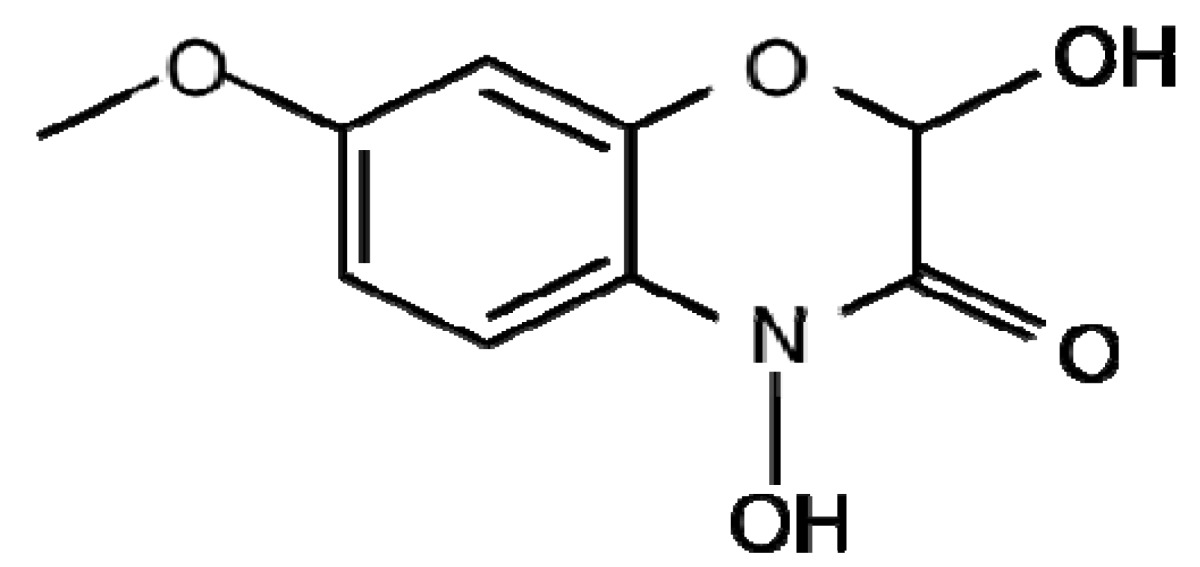	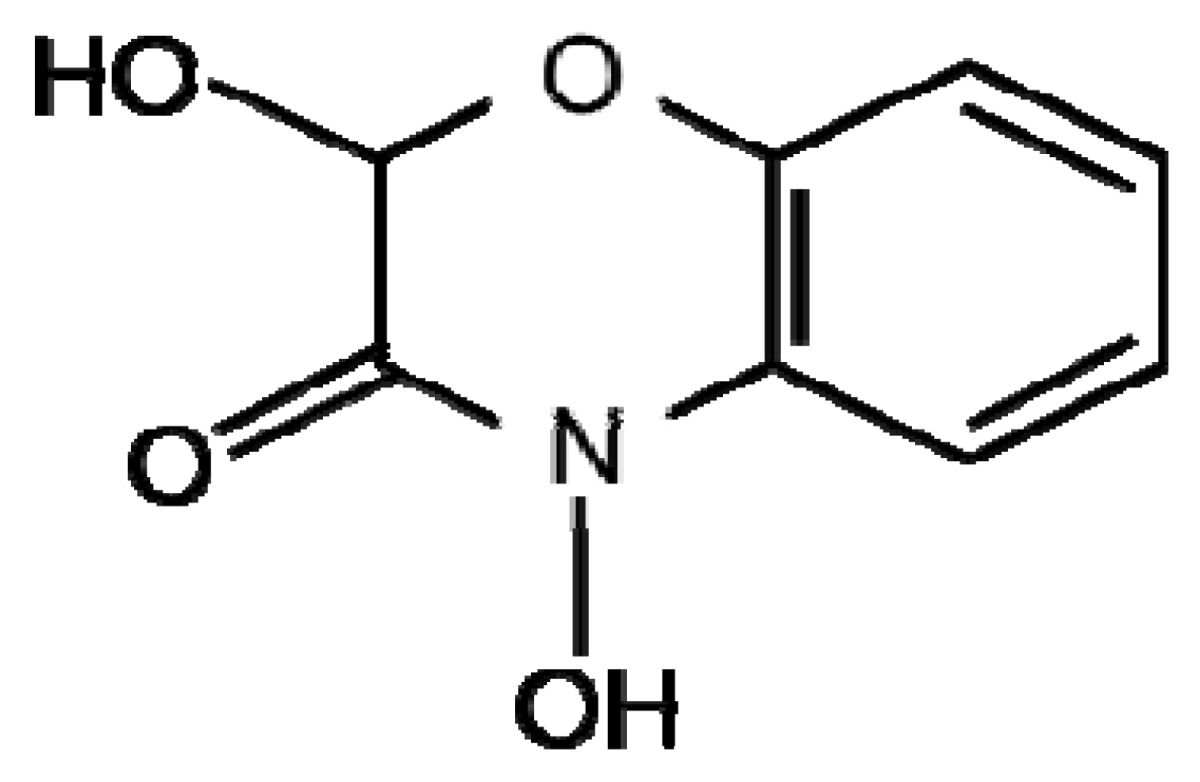
	**DIMBOA**	**DIBOA**
**Terpenoids**	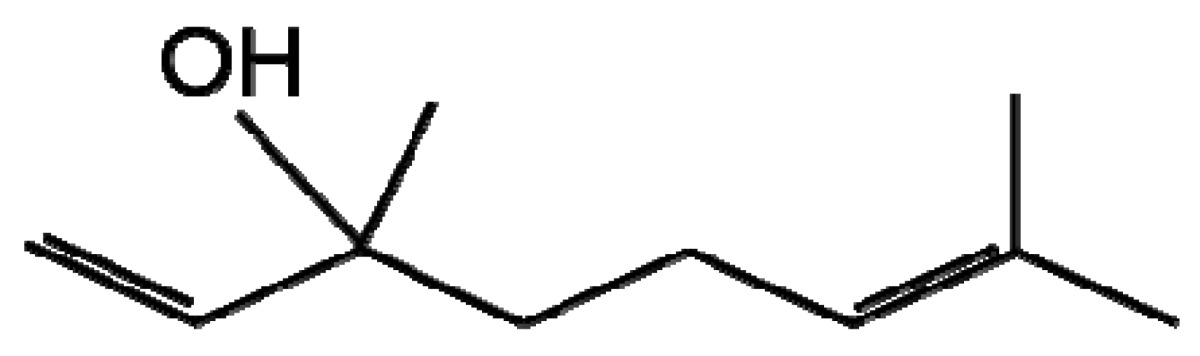	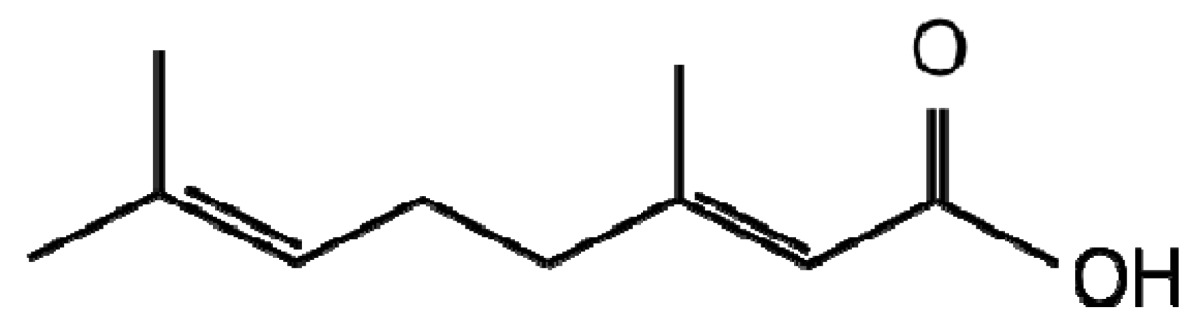
	**Linalool**	**Geranic acid**
**Flavonoids**	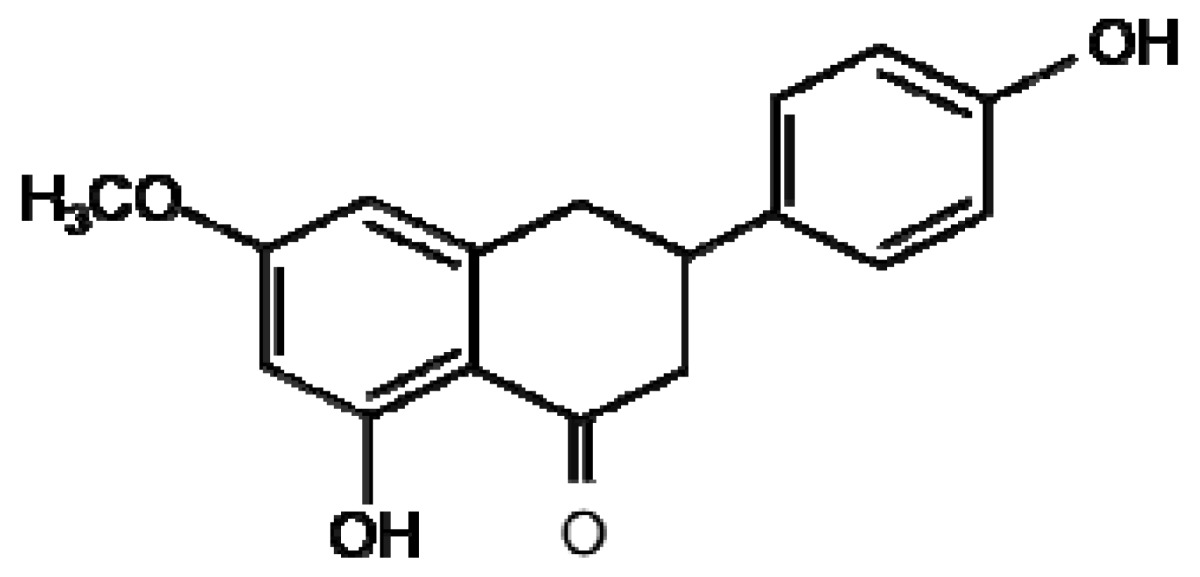	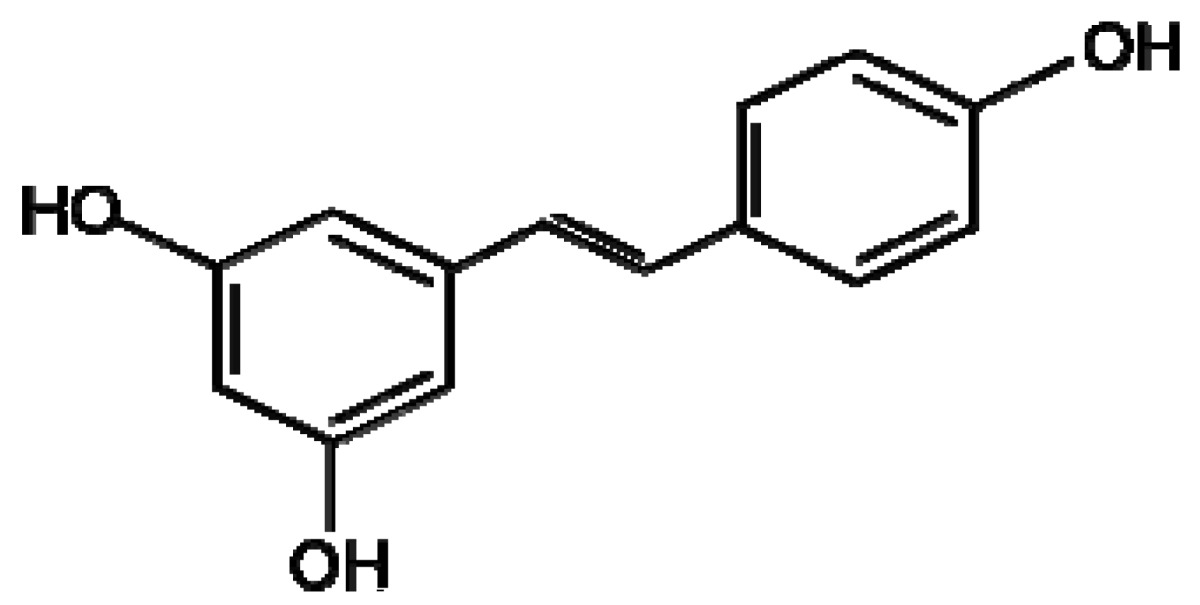
	**Sakuranetin**	**Resveratrol**
**Cyanogenic glycosides**	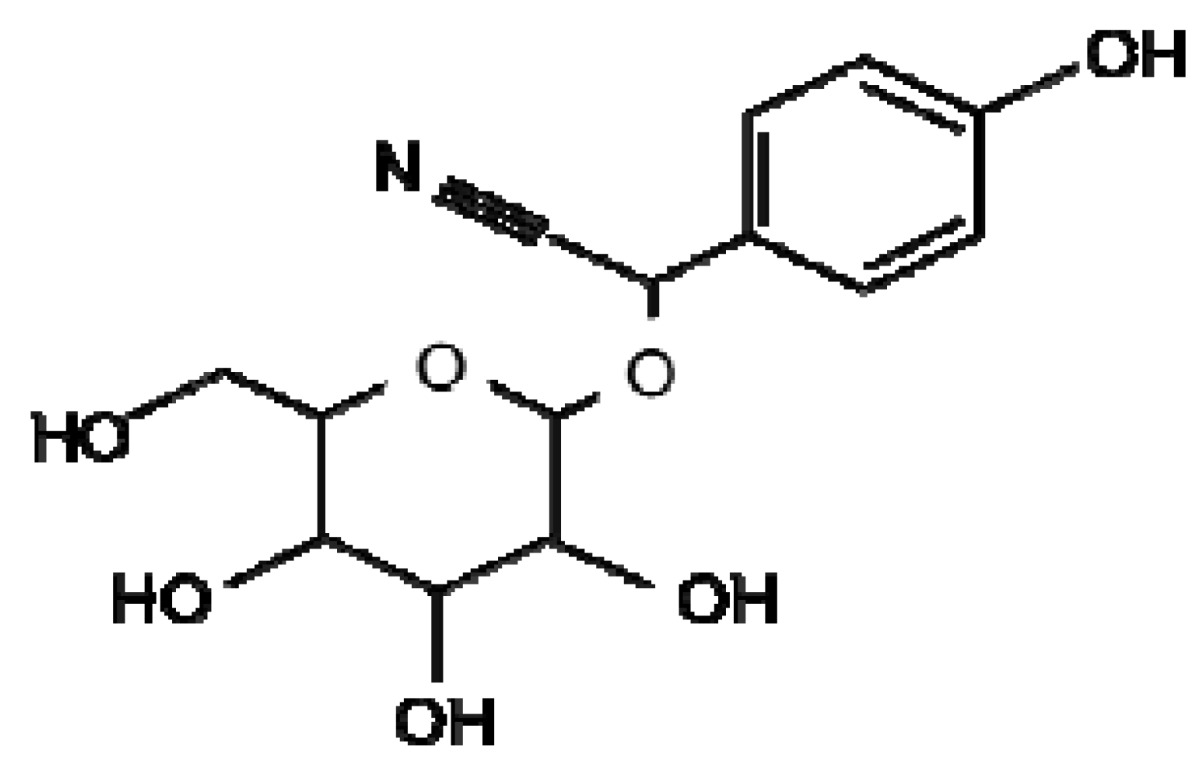	
	**Dhurrin**	
**Saponins**	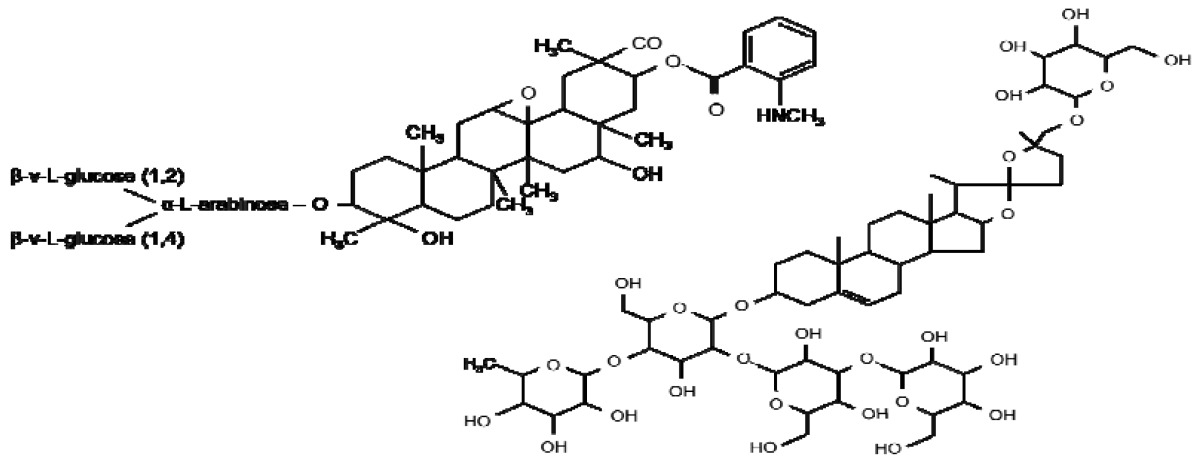	
	**Avenacin A1**	**Avenacoside B**

A recent study used LC-MS/MS to quantify Bxs in 54 Danish wheat varieties discovering the concentration of six Bxs to correlate positively with resistance to Fusarium Head Blight (FHB) [22]. FHB is a destructive disease affecting grain yield and cereal quality and is also capable of producing mycotoxins that can have significant effects on human health.

## 3. Terpenes and Terpenoids

The terpenes and terpenoids are the largest and most diverse class of secondary metabolites with over 40,000 compounds described [23]. Terpenes are synthesised from the basic five-carbon isoprene unit (C_5_H_8_) by the mevalonate or non-mevalonate pathway (Figure 1). The isoprene units are added together via condensation reactions to form branched and cyclised isoprene polymers (hemiterpenes, monoterpenes, sesquiterpenes, diterpenes, sesterterpenes, triterpenes, tetraterpenes and polyterpenes). Terpenoids were originally defined as oxidised terpenes [24], however the term terpenoid is generally used to encompass both of these classes and will in this review. Terpenoids have an extensive range of important roles in the plant kingdom including functioning as plant hormones, electron carriers, vitamins, pigments and membrane components; a number are also involved in plant-pathogen interactions [25]. Terpenoids are produced in various cellular organelles often restricted to specific tissues where activity is required and they are stored in specialised secretory or glandular structures protecting the host plant from potential toxicity of the compounds [25,26]. The presence of a large number of terpenoid compounds in rice has been positively correlated with reduced pathogen infection [27]. Two diterpenoids produced in rice leaves upon *Magnaporthe grisea* infection, momilactones A and B have received particular attention for their antifungal activity against this fungus, the casual agent of the devastating rice blast disease [27-29]. Another group of similar diterpenoids named oryzalexin A–D were identified as rice phytoalexins also in *M. grisea* infected leaves [30-33]. Later, orzyalexin S and orzyalexin E and F were discovered as additional diterpenoids with potent antifungal activity [34-37]. Five cassane diterpenoids phytocassane A-E were found to increase upon *M. grisea* infection and present at higher concentrations in resistant strains in addition to having antifungal activity against another pathogenic fungus *Rhizoctonia solani* [38].

A recent study collected volatile organic compounds (VOCs) released by oat, barley and wheat in response to infection by three *Fusarium* species including two species that cause cortical rot disease of wheat. Piesik *et al.*, measured the VOCs using GC-MS identifying two terpenes linalool (Figure 1) and β-caryophyllene to be present at higher concentrations in infected tissue than controls [39]. The same authors carried out a similar study in maize identifying three additional terpenes induced upon infection, β-pinene, β-myrcene and Z-ocimene [40]. A substantial amount of research into linalool synthesis and natural production has been undertaken due to its aroma and flavour in flower and vegetables for the application of perfume manufacture to metabolic engineering in tomatoes [41,42]. However, little is known regarding its involvement in plant pathogen interactions and the mechanism is assumed to be similar to other terpenoids for which evidence suggests interference and disruption of membranes [43-46]. Piesik *et al.* also demonstrated the ability of infected plants to lead to an increase in VOCs in uninfected neighbours. Control of VOC release in plants has significant potential for the management of crop pathogens. An early study of volatiles in wheat showed it contained the same major terpenoid species as oat and barley [47]. The utility of recent technological advances analysing VOCs using solid phase microextraction (SPME) and headspace techniques for the analysis of terpenes and other volatiles has been demonstrated [48].

Investigation into terpenoids with antifungal activity against two maize pathogens *Fusarium graminearum* and *Colletotrichum graminicola* identified geranic acid (Table 1), which had a minimal inhibitory concentration of 7.8 μg/mL and is the most potent antifungal towards these two pathogens discovered [49]. In an attempt at metabolic engineering to increase resistance of maize to these pathogens, the enzyme geraniol synthase was cloned and overexpressed. Non-targeted LC-MS metabolite profiling and volatile GC-MS headspace analysis identified significant differences in the volatile and non-volatile transgenic extracts of all geraniol derivatives. However, this did not translate into an increased resistance to *F. graminearum* or *C. graminicola*, the authors suggesting potential lack of bioavailability or inappropriate localisation that may be corrected by up or down-regulation of other genes involved in the pathway. Despite this, the author's are of the opinion that metabolic engineering of terpenoid metabolism in maize still has potential as the transgenic plants were of normal phenotype unlike previous attempts at terpenoid engineering in tomato, arabidopsis and potato [50-52].

## 4. Flavonoids (Proanthocyanidins, Anthocyanins, Flavonols, Isolflavonoids)

Flavonoids are a large class of phytoanticipan and phytoalexin phenolic metabolites synthesised from phenylalanine in the shikimate pathway (Figure 1) and includes the flavonols, flavones, flavanones, anthocyanidins, proanthocyanidins and chalcones. Flavonoids play an extensive role in many plant processes such as signalling; antioxidant activity, feeding deterrents, antimicrobial activity, UV protection, male fertility and flower pigmentation [53-55]. Flavonoids have received a significant amount of interest due to their potential uses in the pharmaceutical industry due to their anti-inflammatory and anticancer properties [56], however flavonoids also play numerous important roles in plant resistance, defence, signalling and symbiosis [57]. A number of mechanisms of antimicrobial action have been hypothesised for flavonoids including the crosslinking of microbial enzymes, inhibition of cellulases and other microbial enzymes, chelation of metals necessary for microbial enzyme activity and polymerisation into crystalline structures which may act as a physical barrier during pathogen attack [58].

A number of preformed flavonoids (phytoanticipans) belonging to the anthacyanidin class inhibit the growth and spore germination of the fungal and bacterial pathogens of rice *M. grisea* and *Xanthomonas oryzae* [59]. Flavonoid production can also be induced upon pathogen attack, an example of flavonoid phytoalexins are 3-deoxyanthocyanidin flavonoids induced in Sorghum by *C. graminicola* [60]. These secondary metabolites inhibit fungal growth *in vitro* and are induced during the initial stages of infection only in cells in direct contact with the fungus. The flavonoid sakuranetin (Figure 1) was identified using LC-MS to be induced following treatment of rice with the fungal elicitor chitosan [61]. Proanthocyanidins have been demonstrated to play a part in defence against *Fusarium* species through suggested mechanisms such as chelation of metals required for enzymatic activity, formation of a physical barrier, inhibition of cellulases and crosslinking of microbial enzymes [58]. A number of recent metabolomics studies employing an orbitrap LC-MS instrument have investigated barley resistance to Fusarium head blight identifying 16 flavonoids or isoflavonoids that were resistance related [62,63]; these compounds are yet to be tested for direct antifungal activity. LC-MS/MS analysis was used to identify two new flavonoid phytoalexins induced in response to inoculation of a resistant and susceptible cultivar of sorghum with *Colletotrichum sublineolum* [64]. Luteolin and apigenin were both present at higher concentrations in these cultivars suggestive of a phytoalexin role. Fungal germination bioassays indeed found luteolin to strongly inhibit fungal growth and spore germination; effects were similar but less dramatic for apigenin.

A number of flavonoid compounds require compartmentalisation in the cell to avoid mutagenic and oxidative effects of the active compounds and intermediates in their synthetic pathways. In maize, barley and rye a number of different mechanisms of vacuolar import have been identified including a vacuolar ATP-binding cassette (ABC) transporter, multidrug resistance-associated protein like ABC transporter and pH-dependent vacuolar flavonoid/H+ antiporters [65-68]. The synthesis of the flavone saponarin in mesophyll protoplasts without vacuoles was inhibited indicating that a functioning vacuole is critical for production of this flavone [69]. Flavonoids have recently been the subject of investigation into metabolic engineering of crop plants for the purposes of disease resistance to health benefits for humans [70]. Transgenic wheat and barley were constructed expressing a stilbene synthase gene from *Vitis vinifera* (Common Grape Vine) resulting in the production of the phytoalexin resveratrol (Figure 1) [71]. The authors present results detailing increased resistance of wheat and barley producing resveratrol to the necrotrophic pathogen *Botrytis cinerea*.

## 5. Cyanogenic Glycosides

Cyanogenic glycosides are present in over 2,600 plant species and a number of cereals including wheat, barley, oats, rye, sorghum, millets, sugar cane, maize and rice [72]. These compounds are derived from the amino acids valine, leucine, isoleucine, phenylalanine or tyrosine and the non-protein amino acid cyclopentenyl-glycine as path of the shikimate pathway (Figure 1) [73]. To avoid toxic release of hydrogen cyanide (HCN) under normal conditions, cyanogenic glycosides are compartmentalised within cells separated from the HCN releasing β-glucosidases. Cyanogenic glycosides are activated by β-glucosidase-dependent hydrolysis to form the unstable aglycone upon tissue disruption. This cyanohydrin is further enzymatically (hydroxynitrile lyase) or spontaneously (at alkaline pH) converted to a ketone or an aldehyde and the toxic constituent of the compound, HCN [74,75]. Cyanide is toxic to cells inhibiting the oxidative function of mitochondria cytochrome oxidase thereby reducing the cells ability to use oxygen for aerobic respiration [76,77]. The cyanogenic glycoside dhurrin (Figure 1) found in Sorghum is only located in the epidermal layers of the leaf while the β-glucosidases and α-hydroxynitrile lyase enzymes capable of activation and release of HCN were located only in mesophyll tissue [78].

There is evidence that the production of cyanogenic glycosides occurs at a significant cost to the plant suggesting they must play a role in survival against herbivores or pathogens [79]. Some fungi are capable of detoxifying HCN [80] while others are capable of cyanide-resistant respiration [81]. While there is indisputable evidence for a role of cyanogenic glycosides as herbivore deterrents [72,82], there is little reliable evidence for direct roles against pathogens [17,83]. Very early studies have related the Fusarium wilt resistance of flax to HCN release in roots [84]. HCN release occurs in leaves of *Lotus corniculatus* upon pathogen invasion arresting the development of most fungal species [84]. A recent study in barley investigated five leucine-derived cyano glycosides however discovered that the β-glucosidase that hydrolyses them is only present in the endosperm of germinating barley therefore concluding that the cyanide potential of barley cannot be harnessed in a fungal attack [73].

High performance liquid chromatography (HPLC) and LC-MS/MS for the analysis and identification of cyanogenic glycosides has recently been exploited for the sensitive detection of these compounds and their derivatives [85-87]. The potential of these techniques should be utilised to confirm the role of these compounds in plant defence.

## 6. Saponins

Saponins are a class of glycosylated triterpenes; steroids and steroidal alkaloids synthesised from the mevalonate or non-mevalonate pathway in plants (Figure 1) and are absent in the majority of monocotyledon plants and all cereals with the exception of oat (*Avena*). These phytoanticipans possess a broad range of biological activities including antimicrobial, insecticidal, allelopathic action and molluscidal acitivity [17,88]. Oat contains two types of Saponins—Four triterpenoid avenacins and two steroidal avenacosides (Figure 1) present in roots and leaves respectively [89,90]. Avenacins are active in their natural glycoslyated form in the plant in contrast to avenacosides, benzoxazanoids and many other antifungal compounds which are active only in their aglycone forms [91,92]. The inactive avenacosides are stored in the vacuole of the plant and activated when tissue damage caused by pathogenic fungi disrupts membranes allowing the plant enzyme β-glucosidase to hydrolyse the D-glucose unit forming the biologically active aglycone [93]. The active form of the avenacosides then forms complexes with membrane sterols disrupting the fungi's plasma membrane by pore formation resulting in fungal cell death. Avenacins, which are active in their native form, are also stored in the vacuole of plants, which are protected from their toxic effects by a different membrane sterol composition [17,89,94]. In line with this, several fungi are resistant to saponin-producing plants due to their natural membrane composition. The biological activity and biosynthesis of saponins has been recently reviewed [95].

Saponins have been implicated in the resistance of oat to *Gaeumannomyces graminis* var. *tritici* referred to as the ‘take all’ disease causing severe crop losses in saponin deficient barley and wheat [96]. This hypothesised saponin-conferred resistance of oat is supported by the ability of *G. graminis* var. *avenae* to infect oat due to the possession of the saponin-detoxifying enzyme avenacinase [97]. Saponins are induced by elicitors of defence responses such as jasmonate derivatives [98] again emphasising their role in defence.

In the past, research on saponins has proved difficult, relying on HPLC methods or non-specific stains [88] however recent developments in mass spectrometry and metabolite profiling are enabling the high throughput screening and identification of a large number of these secondary metabolites. These techniques are now being employed to ascertain biosynthetic mechanisms of saponins and related compounds in different plant species and have potential to identify new metabolites belonging to this class of compounds [99]. GC-MS has been combined with gene expression analysis to identify a number of genes involved in triterpene synthesis to also be present in rice. Expression of the oxidosqualene cyclase (OSC) enzyme AsbAB1 encoding the β-amyrin synthase in rice showed that rice is capable of β-amyrin synthesis [100] hence identifying the potential for metabolic engineering of saponin regulated resistance in other cereals. A method for the quantification of saponins using LC-MS/MS has recently been developed [101].

## 7. Conclusion

This review has covered the major classes of secondary metabolites present in cereals with important roles in pathogen defence. The majority of these plant secondary metabolites, whether preformed or induced, are compartmentalised within vacuoles or other specialised cellular compartments to avoid self-toxicity. A common mechanism of activation is enzymatic hydrolysis following vacuole disruption during tissue damage caused by the pathogen. Other compounds accumulate in the apoplast such as benzoxazanoids, which act as defence regulatory signals. Volatile secondary metabolites are also involved in pathogen defence with a number of volatile terpenoids demonstrated to increase in response to pathogen attack. Infected plants are also capable of stimulating volatile release from uninfected neighbouring plants, a feature that may be invaluable to increasing crop resistance to pathogens. The mechanism of action of the antimicrobial secondary metabolites discussed in this review varies from membrane disruption and pore formation (saponins and terpenoids) to interference with aerobic respiration (cyanogenic glycosides) and inhibition of microbial enzymes, chelation of metals required for microbial enzymes and polymerisation forming crystalline physical defence barriers (flavonoids). Microbes are constantly evolving mechanisms to overcome the activity of such compounds as are plants evolving new defence mechanisms. If the potential of metabolite engineering is harnessed, cereals may not be limited to only the classes of secondary defence compounds discussed in this review.

The discovery of secondary metabolites with roles in pathogen defence has been catalysed in recent years with technical advances in mass spectrometry and high throughput metabolite profiling. Many of the metabolites described in this review have been identified via gas chromatography and headspace analysis of volatiles coupled to mass spectrometers in addition to liquid chromatography-mass spectrometry and occasionally nuclear magnetic resonance spectroscopy. The current bottleneck in these techniques is the processing of the large data sets generated and positive identification of all the compounds analysed.
